# Estimating the optimal threshold for a diagnostic biomarker in case of complex biomarker distributions

**DOI:** 10.1186/1472-6947-14-53

**Published:** 2014-06-14

**Authors:** Fabien Subtil, Muriel Rabilloud

**Affiliations:** 1 Université de Lyon, F-69000 Lyon, France; 2 Université Lyon 1, F-69622 Villeurbanne, France; 3 CNRS, UMR5558, Laboratoire de Biométrie et Biologie Evolutive, F-69622 Villeurbanne, France; 4 Hospices Civils de Lyon, Service de Biostatistique, 162 avenue Lacassagne, F-69003 Lyon, France

**Keywords:** Sensitivity and specificity, Diagnostic marker, Optimal cut-point, Semi-parametric Bayesian method

## Abstract

**Background:**

Estimating the optimal threshold (and especially the confidence interval) of a quantitative biomarker to be used as a diagnostic test is essential for medical decision-making. This is often done with simple methods that are not always reliable. More advanced methods work well but only for biomarkers with very simple distributions. In fact, biomarker distributions are often complex because of a natural heterogeneity in marker expression and other heterogeneities due to various disease stages, laboratory equipments, etc. Methods are required to estimate a biomarker optimal threshold in case of heterogeneity and complex distributions.

**Methods:**

A previously described Bayesian method developed for normally distributed biomarkers is applied to two flexible distributions; namely, a Student-*t* and a mixture of Dirichlet processes. Here, numerical studies assess the adequacy of the previous method with both distributions. Two applications are presented: the diagnosis of treatment failure after prostate cancer treated by ultrasound and the early diagnosis of cancers of the upper aerodigestive tract.

**Results:**

Bayesian inference provided reliable credible intervals in terms of bias and coverage probability. The two distributions analysed gave meaningful clinical interpretations in both applications.

**Conclusions:**

Reliable methods can be used to estimate a biomarker optimal threshold, even in case of complex distributions.

## Background

Nowadays, more and more biomarkers are used for screening, diagnosis, prognosis, or monitoring. Some well-known examples are the prostate-specific antigen (PSA) in prostate cancer [1] and all novel proteomic and genomic markers [2,3]. The comparison of the diagnostic accuracy of a set of continuous biomarkers is generally performed through the comparison of their Receiver Operating Characteristic (ROC) curves [4]. A ROC curve reflects the ability of a marker to classify correctly the tested subjects regardless of a specific threshold; it is thus helpful in choosing the most discriminating biomarker. However, after this choice, a threshold value must be determined before the use of the marker as a diagnostic test.

The optimal threshold is defined as the one that leads to the highest sum of test sensitivity and specificity (within this sum, different weights may be given to sensitivity and specificity). The sensitivity and the specificity are often estimated from the empirical distribution function of the biomarker in diseased and non-diseased individuals but this method is not always the best one in terms of bias or root mean square error [5]. Moreover, with this method, only a bootstrap confidence interval is available though the bootstrap coverage probability is not always acceptable for optimal thresholds [6]. Other non-parametric or semi-parametric methods have been proposed; they are based on the smoothing of the ROC curve using loess procedures [7] or on the parametric smoothing of the ROC curve [8]; but, here again, the confidence interval is based on a bootstrap approach.

More efficient methods assume a given parametric distribution for the biomarker in the diseased and non-diseased group. With some specific distributions (e.g., normal, log normal, gamma, or normal after Box-Cox transformation), the optimal threshold is given by an explicit formula that depends on the distribution parameters, and the threshold's confidence interval can be obtained using the delta method [5,6]. However, all biomarkers do not always follow such simple distributions. There are often outlying measurements and the measurements may be obtained with different laboratory equipments having various precisions, which lead to a mixture of distributions with different variances. The presence of various disease stages may also lead to a mixture of distributions with different means. In such cases, there is no explicit formula for the optimal threshold. An estimate can be obtained by numerical algorithms, but the delta method is no more applicable to build the confidence interval.

This article relies on a Bayesian method that estimates the optimal threshold of a biomarker; however, this method was previously applied only to biomarkers normally distributed [9]. It is here extended to two different distributions able to cope with heterogeneity of the variances or the means: the Student-*t* distribution (particularly useful in case of heterogeneous variances) and a mixture of Dirichlet processes (useful in case of heterogeneous means and variances). Simulations were first performed to assess the adequacy of the proposed methods. The methods were next applied to two examples: the diagnosis of prostate-cancer recurrence and the diagnosis of the cancers of the upper aerodigestive tract.

This article shows that, using appropriate methods for threshold determination, rational medical decisions can be made despite patient heterogeneity.

## Methods

### The Bayesian method to estimate the biomarker optimal threshold and its credible interval

This section clarifies briefly the meaning of “optimal threshold” and summarizes the Bayesian method originally used to estimate this threshold and its credible interval in normally distributed biomarkers [9]. By convention, it will be assumed that high values of the marker are indicative of the presence or the exacerbation of the disease.

### Determination of the optimal threshold using the decision theory

The optimal threshold is often defined as the value that best separates the two biomarker distributions relative to the diseased and non-diseased subjects; i.e., the value that maximizes the sum of sensitivity plus specificity (also called the Youden index). The benefits of a good classification and the costs of a misclassification in terms of health status as well as the prevalence of the disease can also be taken into account [10]. For example, for a screening test of a low-prevalence disease, a low threshold should be chosen to favour specificity. This leads to a generalized Youden index that gives unequal weights to sensitivity and specificity:

(1) $$U\left(c\right)=\mathrm{Sen}\left(c\right)+\mathrm{Spe}\left(c\right)\times R,\;R=\frac{\mathrm{NC}}{\mathrm{NB}}\times \frac{1-\pi }{\pi }$$

*NB*/*NC* is the ratio of the net benefit of detecting a diseased subject to the net cost of mistakenly classifying a healthy subject as diseased [11], and *π* is the prevalence of the disease.

### Bayesian optimal-threshold estimation

Maximizing the Youden (or generalized Youden) functions requires the estimation of sensitivity and specificity at different thresholds; hence, knowing the distributions of the marker values in diseased and non-diseased subjects. Parametric distributions are assumed in diseased and non-diseased subjects. In a Bayesian paradigm, priors are defined for the parameters of these parametric distributions, possibly non-informative. The posterior distributions of these parameters can be obtained using Bayes theorem. It is assumed that *K* values of these parameters can be sampled from their posterior distributions, possibly using MCMC algorithms [12]. A Youden function is defined for each of these *K* values and maximized using a numerical method such as the Newton–Raphson method. The values obtained for each of the Youden functions form a sample from the posterior distribution of the optimal threshold. The mode, the mean, or the median of this distribution provides an estimate of the optimal threshold. A 1 − *α* credible interval can be obtained using the empirical *α*/2 and 1 − *α*/2 quantiles of this distribution (the quantile method) or the highest posterior density (HPD) region [13].

Originally applied only to normally distributed biomarkers, this method can be used regardless of the distributions of the marker values in the diseased and non-diseased subjects, with possibly different kinds of distributions. The sole constraint is being able to sample from the posterior distribution of the parameters of these distributions. However, this method may not be robust to a misspecification of the distributions of the marker values, especially when the measurement heterogeneity is high in one group or in both groups. The following sections describe two flexible distributions able to deal with heterogeneity: the Student-*t* distribution (used with heterogeneity in the variance of the measurements) and a mixture of Dirichlet processes (used with heterogeneity in the mean and the variance of the measurements).

### Heterogeneity in the variance of biomarker measurements

#### Optimal threshold with Student-*t* distribution

When measurements are repeated in subject, for example a non-diseased subject, a variation of the marker value might be observed due to intra-subject variability. This variation may follow a normal distribution; however, in the whole non-diseased population, the distribution of the marker may not be normal because that intra-subject variability may itself vary between subjects. Another source of heterogeneity in the variance is the fact that measurements are analysed by different laboratories having different equipment precisions. The marker distribution in each laboratory may be Gaussian, for example, in non-diseased subjects, but non-Gaussian over all laboratories due to precision variability.

In the case of heterogeneity in the variance of the measurements, a Student-*t* distribution might be used (instead of a normal one) because it can be considered as a mixture of an infinite number of normal laws centred around the same value but with a variance distributed according to the inverse of a chi-square distribution. Hence, the Student-*t* distribution may deal with the heterogeneity of the variance among subjects or laboratories. This is also and mainly a robust alternative to the use of a normal distribution in presence of outliers due to measurement errors, because the Student-*t* distribution has longer tails than its normal counterpart.

Let (*μ*, *σ*^2^, *ν*) denote the parameters of the Student-*t* distribution (mean, variance, degrees of freedom, respectively). Non-informative prior distributions might be used for *μ* and *σ*^2^, such as a normal distribution centred on zero with high variance for *μ* and a uniform distribution for log(*σ*). For 1/*ν*, a uniform distribution between 0 and 1 can be chosen as proposed by Gelman et al. [14]. Efficient methods to sample from the posterior distribution of the parameters of a Student-*t* distribution using the Gibbs sampling algorithm are given by Gelman et al. [14]. Software WinBUGS can be used to perform those MCMC calculations [15] (an example is given in the Additional file 1). The Youden function is then calculated for each sample value using the cumulative distribution function of the Student-*t* distribution and maximized using a numerical method such as the Newton–Raphson method. This leads to the posterior distribution of the optimal threshold.

#### Simulations

Three types of simulations were performed to test the proposed method, with different marker distributions: a true normal law, a Student-*t* law, or a mixture of two normal laws with different variances. In each case, the relative bias of the optimal threshold estimate stemming from the mean, the median, and the mode of the posterior distribution were calculated, as well as the mean width, and the coverage probability of the 95% credible interval obtained with the quantile or the HPD method. The calculations were performed over 5 000 simulated data sets. The *NB*/*NC* ratio was set to one, and the prevalence to 0.5. The theoretical optimal threshold was calculated using explicit formulae or the Newton–Raphson algorithm. For comparison, the optimal threshold was also estimated using the empirical boxcox, and kernel methods proposed by Fluss et al. [5], and, in each case, the associated bootstrap 95% confidence interval.

##### Marker values truly distributed according to a normal law (Design 1)

Marker values were generated from a normal distribution, with mean −0.3 and standard deviation *σ*_0_ in *N*_0_ non-diseased subjects, and mean −0.2 and standard deviation *σ*_1_ in *N*_1_ diseased subjects. The posterior distribution of the optimal threshold was estimated assuming a Student-*t* distribution, then a normal distribution in each group. The following parameter values were used: *N*_0_ = *N*_1_ = {50, 100} and *σ*_0_ = *σ*_1_ = {0.03, 0.05, 0.07}. Another set of simulations was performed with unequal standard deviation: (*σ*_0_, *σ*_1_) = {(0.07, 0.03), (0.03, 0.07)}. Results are also given in the Additional file 2 with unequal sample sizes: (*N*_0_, *N*_1_) = {(100, 50), (100, 75)}. The standard deviation values were chosen lower than the difference between the means of the two groups to prevent a total overlap of marker distributions. The sample sizes were chosen to be of the same magnitude as those found in diagnostic studies.

##### Marker values truly distributed according to a Student-t law (Design 2)

Marker values were generated from a normal distribution in the non-diseased group, with mean −0.3 and standard deviation 0.05, then from a Student-*t* distribution in the diseased group, with mean −0.25, standard deviation 0.05, degrees of freedom *ν*, and 100 subjects in each group. Four values of *ν* were used: {1, 4, 8, 12}, the first corresponded to a distribution with a lot of outliers and the last to a distribution similar to the normal one. The posterior distribution of the optimal threshold was estimated assuming first a Student-*t* distribution then a normal distribution in each group.

##### Marker distributed according to a mixture of two normal laws in the diseased subjects (Design 3)

Marker values were generated from a normal distribution in the non-diseased group, with mean −0.3 and standard deviation 0.05, and from a mixture of two normal distributions in the diseased group, both centred on −0.25 but one with a standard deviation of 0.05 and the other, associated only to a proportion *p* of subjects, with a standard deviation of *σ*_2_. One hundred diseased values and one hundred non-diseased values were generated. In the diseased group, the mixture reflects the heterogeneity in measurements (heterogeneity attributed to subjects or to lab equipments). It can also reflect the presence of a proportion *p* of outlying measurements, with over dispersion. The following parameter values were used: *σ*_2_ = {0.075, 0.10} and *p* = {0.1, 0.2, 0.3}. For smaller values of *σ*_2_, the two distributions in the diseased group would be indistinguishable.

#### Application

The above-described method was applied to a study involving 289 subjects followed-up after a high-intensity focused ultrasound (HIFU) treatment for prostate cancer. The treatment was delivered at Edouard Herriot hospital (Lyon, France) between 2000 and 2007. The follow-up consisted of biopsies and regular PSA measurements. Among these subjects, 150 had one positive biopsy during follow-up and were considered as cases of treatment failure or cases of prostate cancer recurrence (diseased subjects); the remaining 139 who had no positive biopsy were considered as non-diseased subjects.

According to the current French Legislation, an observational study that does not change routine management of patients does not need to be declared or submitted to the opinion of a research ethics board.

**Figure 1:**
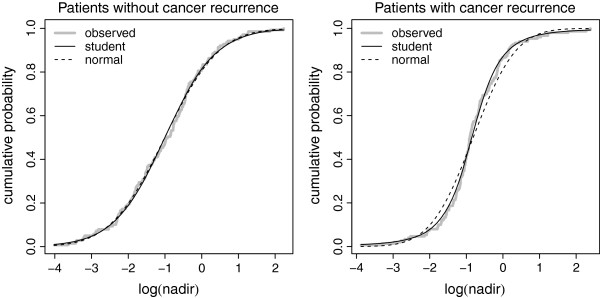
**Cumulative distribution of the PSA nadir.** Cumulative distribution of the PSA nadir in subjects with and without prostate cancer recurrence, along with the predicted cumulative distributions using a normal or a Student-*t* distribution.

The PSA nadir (i.e., the lowest PSA measurement during a given subject follow-up) was used as a good criterion to detect treatment failure, and hence to trigger biopsies [1]. A threshold is required to use this criterion as a diagnostic test. The distribution of the logarithm of the PSA-nadir in non-diseased subjects was not found to depart from a normal distribution (Figure 1) but this was not the case in the diseased subjects because of some outlying log-PSA nadirs.

A Student-*t* distribution was assumed in both groups and 2000 samples from the posterior distribution of the parameters of the Student-*t* law in each group were considered to estimate the optimal PSA-nadir threshold. The prevalence was fixed to 52%, which corresponds to the prevalence observed in the prospective study. In this example, the *NB*/*NC* value was set set to 1.5 (equivalent to triggering biopsies above 40% probability of treatment failure).

### Heterogeneity in the mean and the variance of biomarker measurements

#### Optimal threshold using a mixture of Dirichlet processes

Among diseased subjects, there may be an inter-subject heterogeneity in marker measurements due to various stages of the disease. When this heterogeneity is linked to factors such as age or sex, these factors may be taken into account and one marker threshold may be proposed for each factor combination. However, heterogeneity may be linked to factors unknown at the time of marker measurement such as the stage or the severity of the disease. Hence, when the marker is normally distributed at each stage of the disease, the marker measurements over all diseased subjects are the result of a mixture of normal distributions with different means and different variances.

In this section, the distribution of the marker measurements in each group was modelled by a mixture of normal laws, with means and variances distributed according to a Dirichlet process (*G*), which led to a mixture of Dirichlet processes [16,17]. Dirichlet processes are used in the Bayesian context and can be seen as mass-point distributions. They are characterized by two parameters: *G*_0_, which can be considered as a prior distribution for *G*, and *M* that characterizes the degree of belief in *G*_0_. Hence, the number and location of the mass-points in *G* are estimated from the data and from the prior distributions. Parameter *M* can be estimated from the data using a Gamma prior as suggested by Escobar and West [17]. The full specification of the model is given in the Additional file 3, as well as the sampling techniques. Globally, all priors chosen were uninformative or little informative.

The mixture of Dirichlet processes belongs to the class of semi-parametric methods in Bayesian modelling and are flexible enough to fit a lot of distributions, especially mixture of distributions.

#### Simulations (Design 4)

When the true distribution of the marker is normal in diseased and non-diseased subjects, there is no loss of performance using a mixture of Dirichlet processes instead of a normal distribution in terms of relative bias, coverage probability, and mean width of the credible interval (data not shown).

The properties of the method described in this section were assessed in the case of marker measurements distributed according to a mixture of two normal laws in the diseased group (0.5 × *N*(0.05, *σ*_1_^2^) + 0.5 × *N*(−0.25, *σ*_2_^2^)), and a simple normal law in the non-diseased group (*N*(−0.3, 0.07^2^)). *N* marker values were generated in each group. The accuracy of the method was calculated over 5 000 simulated data sets. The following parameter values were used: *N* = {30, 50, 100, 200}, *σ*_1_ = {0.07, 0.08, 0.10}, and *σ*_2_ = {0.05, 0.07}. These standard deviations were chosen according to the means so that a clear mixture of distributions can be identified in the diseased group. The results obtained using a single normal distribution to model the biomarker distribution in the non-diseased group and also a single normal distribution for the diseased group are also reported. The *NB*/*NC* ratio was set to one, and the prevalence to 0.5. For comparison, the optimal threshold was also estimated using the empirical boxcox, and kernel methods proposed by Fluss et al. [5], and, in each case, the associated bootstrap 95% confidence interval.

#### Application

The method described in this section was applied to the diagnosis of cancers of the upper aerodigestive tract using Cyfra 21–1, a tumour marker. A case–control study was conducted between January 1997 and October 2002 in the Department of Otolaryngology-Head and Neck Surgery (Lyon-Sud hospital, Lyon, France). It included 300 patients treated for a cancer of the upper aerodigestive tract and 71 voluntary blood donors assumed to be in good health and free from cancer or inflammatory diseases of the upper aerodigestive tract. The aim was to estimate Cyfra optimal threshold for the early diagnosis of those cancers.

**Figure 2:**
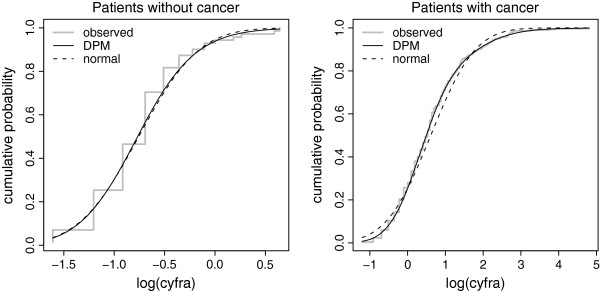
**Cumulative distribution of Cyfra 21–1 values.** Cumulative distribution of Cyfra 21–1 values in subjects with and without a cancer of the upper aerodigestive tract along with the predicted cumulative distributions using a normal distribution or a mixture of Dirichlet processes.

**Figure 3:**
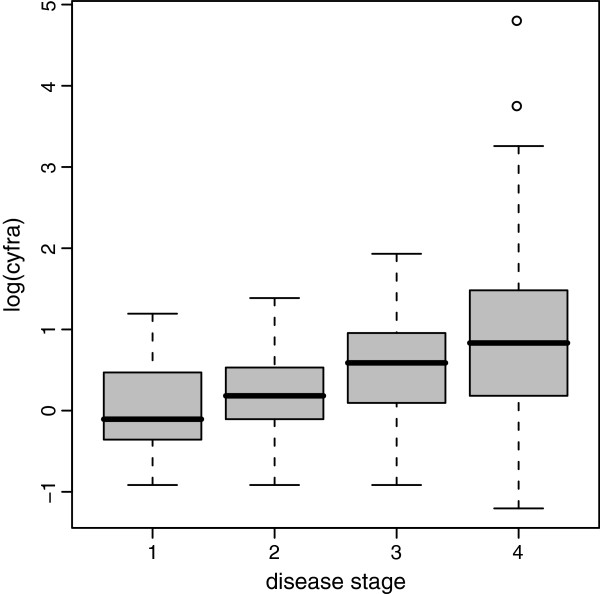
**Boxplots of the logarithms of Cyfra 21–1 values according to the cancer stage.** Boxplots of the logarithms of Cyfra 21–1 values in subjects with a cancer of the upper aerodigestive tract according to the cancer stage.

Here too, according to the current French Legislation, this did not need the opinion of a research ethics board.A normal distribution fitted fairly the distribution of the logarithms of Cyfra values in non-diseased subjects (Figure 2) but not in diseased subjects. The diseased subjects had various disease stages: stage 1 localized cancers, stages 2 and 3 cancers with lymph node invasion, and stage 4 cancers with distant metastases. The distributions of Cyfra values in each stage differed by their means and variances (Figure 3); hence, the distribution over all diseased subjects was the result of a mixture of distributions.

A mixture of Dirichlet processes was used to model Cyfra distribution in diseased and non-diseased subjects. To calculate the posterior distribution of the Cyfra’s optimal threshold, 3 000 iterations of the Gibbs sampling algorithm were considered. The *NB*/*NC* ratio was set by the clinicians to 4 and the prevalence was considered to be 8/1000, an estimate of the French prevalence of cancers of the upper aerodigestive tract [18].

## Results

### Heterogeneity in the variance of biomarker measurements

#### Simulations

This section describes the simulations results regarding the use of the Student-*t* distribution instead of the normal one in case of heterogeneity in the variance of biomarker measurements. The coverage symmetry of the credible intervals for all simulation designs is given in the Additional file 4, as well as the values of the optimal threshold and the Youden index.

##### Marker values truly distributed according to a normal law (Design 1)

In all simulations with equal variances and sample sizes in each group, the relative bias was lower than 0.1% and had the same order of magnitude with both the normal and Student-*t* distributions (Table 1). In case of unequal variances or unequal sample sizes, the relative bias was higher with the Student-*t* than with the normal distribution, but remained lower than 0.75%. The optimal thresholds were generally similar whatever the estimate kept from the posterior distribution (mode, median, or mean). The coverage probability of the 95% credible intervals were around 95% and similar with both distributions and both calculation methods (quantile or HPD). The credible interval mean widths were also similar and decreased with the increase in sample size. The credible intervals were symmetric with the normal distribution, but generally asymmetric with the Student-*t* distribution, except in case of equal variances and sample sizes. Among the other methods tested (Additional file 2), the boxcox method gave similar results to the normal and Student-*t* methods in terms of relative bias and coverage probability of the 95% confidence interval. The empirical method gave more biased results, with a coverage probability farther from 95% than the other methods.

**Table 1:** Simulation results for Design 1

				Relative bias^*^						**Coverage probability**^**†**^				**CI mean width**^**†**^			
				**Mode**		**Median**		**Mean**		**Quantile**		**HPD**		**Quantile**		**HPD**	
***N***_**0**_	***N***_**1**_	***σ***_**0**_	***σ***_**1**_	**Gauss**	***t***	**Gauss**	***t***	**Gauss**	***t***	**Gauss**	***t***	**Gauss**	***t***	**Gauss**	***t***	**Gauss**	***t***
100	100	0.07	0.07	−0.00022	−0.00014	−0.00003	−0.00016	−0.00003	−0.00018	0.945	0.948	0.943	0.944	0.022	0.021	0.022	0.021
100	100	0.05	0.05	0.00006	0.00015	−0.00003	0.00021	−0.00003	0.00021	0.947	0.951	0.945	0.944	0.014	0.015	0.014	0.015
100	100	0.03	0.03	−0.00019	0.00010	−0.00020	0.00016	−0.00020	0.00011	0.948	0.956	0.945	0.951	0.010	0.011	0.010	0.011
50	50	0.07	0.07	0.00124	0.00114	0.00114	0.00106	0.00115	0.00104	0.949	0.953	0.948	0.950	0.032	0.031	0.032	0.031
50	50	0.05	0.05	0.00004	0.00030	0.00002	0.00031	0.00002	0.00030	0.950	0.951	0.949	0.948	0.020	0.021	0.020	0.022
50	50	0.03	0.03	−0.00038	0.00071	−0.00042	0.00072	−0.00043	0.00070	0.944	0.941	0.940	0.940	0.015	0.016	0.015	0.016
100	100	0.07	0.03	0.00047	0.00567	−0.00047	0.00518	−0.00015	0.00530	0.949	0.941	0.950	0.934	0.012	0.013	0.012	0.013
100	100	0.03	0.07	−0.00067	−0.00544	0.00025	−0.00492	−0.00006	−0.00510	0.946	0.938	0.946	0.935	0.012	0.013	0.012	0.013
50	50	0.07	0.03	0.00057	0.00727	−0.00120	0.00608	−0.00055	0.00651	0.948	0.944	0.946	0.940	0.018	0.019	0.018	0.019
50	50	0.03	0.07	−0.00114	−0.00719	0.00063	−0.00610	0.00000	−0.00651	0.953	0.948	0.949	0.942	0.018	0.019	0.018	0.019

^*^Relative bias of the mode, the median, and the mean estimates of the optimal threshold.

^†^Coverage probability and credible interval (CI) mean width found with the quantile and the Highest Posterior Density (HPD) region method.

*N*_0_ and *N*_1_: number of non-diseased and diseased subjects – *σ*_0_ and *σ*_1_ : standard deviation of the distribution of the marker in non-diseased and diseased subjects – Gauss: Gaussian distribution – *t*: Student-*t* distribution.

Hence, even when the true distribution of the marker was normal, there was no great loss of efficiency in using a Student-*t* distribution to estimate the optimal threshold in the proposed simulations. More generally, outside the context of optimal threshold estimation, the Student-*t* is often a good alternative to the normal distribution [19].

##### Marker values truly distributed according to a Student-t law (Design 2)

The relative bias of the optimal threshold estimated using the normal distribution decreased along with the increase of *ν* but was always higher than the one obtained with the Student-*t* distribution, even with *ν* = 12 (Table 2). The coverage probability of the credible interval stemming from the normal distribution was insufficient at small values of *ν* but increased closer to 95% with increasing *ν* values, whereas the coverage probability of the credible interval obtained with the Student-*t* distribution was always around 95%. Hence, when the true distribution of the marker is Student-*t* with a small *ν* value, the normal distribution is not suitable to estimate the optimal threshold.

**Table 2:** Simulation results for Design 2

	Relative bias^*^						**Coverage probability**^**†**^				**CI mean width**^**†**^			
	**Mode**		**Median**		**Mean**		**Quantile**		**HPD**		**Quantile**		**HPD**	
***ν***	**Gauss**	***t***	**Gauss**	***t***	**Gauss**	***t***	**Gauss**	***t***	**Gauss**	***t***	**Gauss**	***t***	**Gauss**	***t***
1	0.2905	0.0055	0.2944	0.0103	0.2929	0.0082	0.000	0.937	0.000	0.932	0.033	0.029	0.034	0.030
4	0.0431	0.0029	0.0437	0.0040	0.0435	0.0036	0.530	0.953	0.532	0.955	0.024	0.022	0.024	0.022
8	0.0147	0.0014	0.0149	0.0019	0.0148	0.0017	0.863	0.951	0.868	0.952	0.023	0.021	0.023	0.021
12	0.0086	0.0009	0.0086	0.0013	0.0086	0.0011	0.918	0.951	0.920	0.955	0.022	0.020	0.022	0.020

^*^Relative bias of the mode, the median, and the mean estimates of the optimal threshold.

^†^Coverage probability and credible interval (CI) mean width found with the quantile and the Highest Posterior Density (HPD) region method.

*ν*: Degrees of freedom of the Student-*t* distribution in diseased subjects – Gauss: Gaussian distribution – *t*: Student-*t* distribution.

The boxcox and kernel methods were generally a little more biased than the Student-*t* method (Additional file 2). The empirical method was a little less biased than the Student-*t* method for small *ν* values. The coverage probabilities were generally farther from 95% with the empirical, boxcox, and kernel methods than with the Student-*t* method, and the 95% confidence intervals were a little wider.

##### Marker distributed according to a mixture of two normal laws in the diseased subjects (Design 3)

The relative bias using a Student-*t* distribution was never above 0.2%, but smaller than the one obtained by using a normal distribution (Table 3). In some cases, differences in relative biases were very large. The coverage probability of the credible interval obtained with the Student-*t* distribution was always close to 95%. Using the normal distribution, this was true only when the proportion of subjects with over dispersed marker values was low and that over dispersion limited.

**Table 3:** Simulation results for Design 3

		Relative bias^*^						**Coverage probability**^**†**^				**CI mean width**^**†**^			
		**Mode**		**Median**		**Mean**		**Quantile**		**HPD**		**Quantile**		**HPD**	
***σ***_**2**_	***p***	**Gauss**	***t***	**Gauss**	***t***	**Gauss**	***t***	**Gauss**	***t***	**Gauss**	***t***	**Gauss**	***t***	**Gauss**	***t***
0.10	0.3	0.0280	−0.0011	0.0287	0.0001	0.0285	−0.0004	0.736	0.956	0.734	0.951	0.025	0.024	0.024	0.023
0.10	0.2	0.0211	−0.0011	0.0215	−0.0001	0.0214	−0.0004	0.804	0.957	0.804	0.954	0.024	0.022	0.024	0.022
0.10	0.1	0.0123	0.0005	0.0125	0.0010	0.0124	0.0007	0.889	0.959	0.887	0.955	0.023	0.021	0.023	0.021
0.075	0.3	0.0084	−0.0019	0.0085	−0.0015	0.0085	−0.0017	0.924	0.953	0.923	0.953	0.023	0.022	0.023	0.021
0.075	0.2	0.0062	−0.0010	0.0063	−0.0007	0.0063	−0.0008	0.935	0.952	0.936	0.952	0.023	0.021	0.023	0.021
0.075	0.1	0.0029	−0.0008	0.0030	−0.0007	0.0030	−0.0007	0.947	0.954	0.942	0.953	0.022	0.020	0.022	0.020

^*^Relative bias of the mode, the median, and the mean estimates of the optimal threshold.

^†^Coverage probability and credible interval (CI) mean width found with the quantile and the Highest Posterior Density (HPD) region method.

*σ*_2_: variance of the distribution in the subjects with over dispersion – *p*: proportion of subjects with over dispersion – Gauss: Gaussian distribution – *t*: Student-*t* distribution.

Hence, though the Student-*t* distribution represents a mixture of an infinite number of normal distributions, it gives satisfactory results in estimating the optimal threshold of a marker when that mixture includes only two distributions per group, both centred on the same value. The Student-*t* distribution is also well-suited for markers with normal distributions and outlying measurements, which is not a rare case. Higher values of *σ*_2_ were not tested; however, a standard deviation more than twice as large in one group than in another group may be uncommon within clinical contexts.

Whatever the simulation setting, the threshold estimates were similar with the mode, the median, or the mean of the posterior distribution. The credible intervals were also similar with either the quantile or the HPD method. Hence, the Student-*t* distribution is a robust alternative to the normal distribution. The empirical, boxcox, and kernel methods were generally more biased and their coverage probability was farther from 95% than the Student-*t* method, except for distributions close to a simple normal one (*σ*_2_=0.075) with the boxcox method (Additional file 2).

#### Application: PSA nadir threshold

In the estimation of the optimal threshold of PSA nadir, the Student-*t* distribution seemed to fit the biomarker distribution in subjects with treatment failure (diseased subjects), contrarily to the normal distribution (Figure 1). This can be explained by the presence of outlying PSA-nadir values in this group. With a Student-*t* distribution, the mode, the median, and the mean of the optimal PSA-nadir threshold distribution were 0.152, 0.154, and 0.153, respectively. The 95% credible interval was [0.105, 0.208] with the HPD method and [0.090, 0.198] with the quantile method. With a normal distribution, the mode, the median, and the mean of the optimal PSA-nadir threshold were 0.100, 0.099, and 0.098, respectively. The 95% credible interval was [0.057, 0.145] with the HPD method and [0.053, 0.142] with the quantile method. As mentioned previously, the Student-*t* distribution reproduced better the empirical cumulative distribution of the PSA nadir in the diseased group than the normal distribution, even if the normal distribution was close to the Student-*t* one. But, as shown in this example, a little change in the distribution function can lead to substantial differences in the optimal threshold estimates. The optimal threshold obtained with a mixture of Dirichlet processes led to a sensitivity of 85.5%, 95% CI [80.8%, 89.2%] and a specificity of 28.9%, 95% CI [22.5%, 35.5%]. Using the empirical method, the optimal threshold was 0.170 (95% CI: [0.109, 0.228]), it was 0.098 with the boxcox method (95% CI: [0.045, 0.147]), and 0.161 with the kernel method (95% CI: [0.109, 0.199]). In this example, the boxcox method is not appropriate because it is not possible to find a common mathematical transformation of the biomarker values in the diseased and non-diseased groups. An example of R [20] code to calculate the posterior distribution of such a threshold with Student-*t* distributions is given in the Additional file 1.

This example highlights the interest of the Student-*t* distribution in estimating the optimal threshold of a biomarker in presence of outlying measurements, which is not rare in the field of medicine.

### Heterogeneity in the mean and the variance of biomarker measurements

#### Simulations (Design 4)

The relative bias of the optimal threshold estimated using a mixture of Dirichlet processes was generally smaller than the one obtained using normal distributions, except when *σ*_1_ = *σ*_2_, and decreased with the sample size (Table 4). With the mixture of Dirichlet processes, the bias was always less than 3.5% for *N* between 100 and 200, and less than 6.8% for *N* below 100. There were little differences between the estimations obtained with the mode, the median, or the mean of the posterior distribution, but no indication that one estimator is always better than the others. The coverage probability of the 95% credible interval was always close to 95% with the mixture of Dirichlet processes for *N* between 100 and 200, and far from 95% using normal distributions. There were small differences between the coverage probabilities obtained with the HPD and those obtained with the quantile method. Here again, there was no indication that one method would be always better than the other. The credible intervals were generally asymmetric. The relative bias of the optimal threshold estimated with a mixture of Dirichlet processes was generally smaller than the one obtained with the empirical, the boxcox, or the kernel method, except for *N* = 30 (Additional file 2) and the coverage probability was generally closer to 95% with the former than with the three latter methods.

**Table 4:** Simulation results for Design 4

			Relative bias^*^						**Coverage probability**^**†**^				**CI mean width**^**†**^			
			**Mode**		**Median**		**Mean**		**Quantile**		**HPD**		**Quantile**		**HPD**	
***N***	***σ***_**1**_	***σ***_**2**_	**Gauss**	**Dirichlet**	**Gauss**	**Dirichlet**	**Gauss**	**Dirichlet**	**Gauss**	**Dirichlet**	**Gauss**	**Dirichlet**	**Gauss**	**Dirichlet**	**Gauss**	**Dirichlet**
200	0.07	0.07	0.0392	−0.0294	0.0405	−0.0087	0.0400	−0.0177	0.623	0.947	0.631	0.926	0.020	0.066	0.020	0.069
200	0.08	0.05	0.1657	−0.0064	0.1668	0.0281	0.1664	0.0082	0.000	0.956	0.000	0.943	0.020	0.080	0.020	0.087
200	0.10	0.05	0.1717	−0.0107	0.1728	0.0167	0.1724	0.0023	0.000	0.959	0.000	0.945	0.020	0.069	0.021	0.074
100	0.07	0.07	0.0386	−0.0394	0.0412	−0.0093	0.0403	−0.0223	0.792	0.944	0.797	0.922	0.029	0.082	0.029	0.086
100	0.08	0.05	0.1644	−0.0074	0.1666	0.0340	0.1658	0.0112	0.000	0.960	0.000	0.947	0.029	0.093	0.029	0.100
100	0.10	0.05	0.1713	−0.0118	0.1731	0.0206	0.1725	0.0039	0.000	0.961	0.000	0.948	0.025	0.077	0.026	0.082
50	0.07	0.07	0.0373	−0.0413	0.0425	−0.0063	0.0406	−0.0215	0.879	0.971	0.884	0.952	0.042	0.100	0.041	0.096
50	0.08	0.05	0.1623	0.0007	0.1668	0.0483	0.1651	0.0243	0.017	0.969	0.020	0.960	0.041	0.114	0.041	0.106
50	0.10	0.05	0.1689	0.0032	0.1736	0.0431	0.1719	0.0244	0.012	0.963	0.015	0.962	0.042	0.102	0.042	0.096
30	0.07	0.07	0.0333	−0.0219	0.0426	0.0015	0.0392	−0.0081	0.923	0.982	0.929	0.974	0.055	0.102	0.054	0.098
30	0.08	0.05	0.1576	0.0394	0.1652	0.0705	0.1624	0.0589	0.111	0.961	0.127	0.962	0.054	0.117	0.053	0.111
30	0.10	0.05	0.1643	0.0498	0.1723	0.0679	0.1693	0.0629	0.091	0.944	0.104	0.951	0.055	0.104	0.054	0.100

^*^Relative bias of the mode, the median, and the mean estimates of the optimal threshold.

^†^Coverage probability and credible interval (CI) mean width found with the quantile and the Highest Posterior Density (HPD) region method.

*N*: number of subjects in each group – *σ*_1_ and *σ*_2_: standard deviation of the normal distributions in diseased subjects – Gauss: Gaussian distribution – Dirichlet: mixture of Dirichlet processes.

#### Application: Cyfra 21–1 optimal threshold

In patients with no cancer of the upper aerodigestive tract, a mixture of Dirichlet processes or a normal distribution reproduced well the empirical distribution of Cyfra (Figure 2). In the diseased group, the fit to the data was good, highlighting the ability of the mixture of Dirichlet processes to fit complex distributions due to mixtures. The mode, the median, and the mean of the posterior distribution of the optimal Cyfra 21–1 threshold were 0.844, 0.841, and 0.838, respectively. The 95% credible interval was [0.739, 0.930] with the HPD method, which is quite close to the interval obtained with the quantile method; i.e., [0.738, 0.930]. The threshold obtained led to a sensitivity of 34.0%, 95% CI [30.3%, 38.0%], and a specificity of 99.9%, 95% CI [98.6%, 99.9%]. Let us remember here that the analysis was carried out in the context of a low-prevalence disease where the maximisation of the generalized Youden index leads to a threshold with a high specificity. Using the empirical method, the optimal threshold was 0.700 (95% CI: [0.600, 1.000]); it was 0.792 with the boxcox method (95% CI: [0.725, 0.865]) and 0.769 with the kernel method (95% CI: [0.665, 0.903]).

We note here that the use of Cyfra for the early diagnosis of cancers of the upper aerodigestive tract in the general population is most convenient at stages 1 or 2; subjects at stages 3 and 4 would be already hospitalized. Though the results of this example are not fully clinically applicable, the example was used mostly because Cyfra distribution from stage 1 to stage 4 was not reproducible using standard distributions.

## Discussion

Within the context of estimating the optimal threshold of a biomarker, rational approaches are rather exceptions, mainly because there is no single method to apply whatever the biomarker. Parametric methods provide stable confidence intervals but, up to now, were constrained to very special cases and required homogeneity in marker distributions in diseased and non-diseased subjects. Unfortunately, when it comes to biology, homogeneity is rather an exception.

The present article is an application of a previously published Bayesian method to estimate the optimal threshold of a biomarker to two distributions among the most able to take into account heterogeneity in marker measurements among subjects. The Student-*t* distribution is well-suited when the marker is normally distributed but with different subgroup variances owing, for example, to different lab equipments. This distribution is also a robust solution in presence of outlying measurements, which is a frequent situation. Besides, simulation results showed that the Student-*t* distribution can be used even when the marker value distribution is truly Gaussian; this results sometimes in an acceptable increase in the relative bias (lower than 0.75%). Anyway, the Gaussian distribution is a theoretical concept; moreover, the simulation results have shown the benefit of the Student-*t* distribution over the normal distribution even in case of a little departure from the normal distribution, due for example to outlying measurements. Other methods, such as the empirical, boxcox, or kernel methods [5], do not give better results in this context; they are generally more biased or have coverage probabilities farther from 95% than the Student-*t* method, except in very specific cases. A mixture of Dirichlet processes has also been used to reflect, for example, a mixture of marker values due to different stages or severities of the disease. Rücker and Schumacher [21] have already used mixture of distributions but they fixed the number of distributions within mixtures. The flexibility of a mixture of Dirichlet processes allows coping with a lot of distribution shapes. In cases of a mixture of distributions leading to a multimodal distribution, a mixture of Dirichlet processes gives good results in terms of bias and coverage probability when the sample size is at least 50 per group. The empirical, boxcox, and kernel methods do not outperform the mixture of Dirichlet processes in this case. For sample sizes smaller than 50 per group, the empirical method is less biased than a mixture of Dirichlet processes, but the coverage probabilities are farther from 95% than with a mixture of Dirichlet processes. For the Student-*t* distribution and the mixture of Dirichlet processes, the credible intervals were generally asymmetric, but this is a common problem in estimating optimal thresholds [10].

The Bayesian inference is very useful for optimal threshold estimation because, frequently, due to the distribution used to model the biomarker in the diseased and non-diseased groups, there is no explicit formula for the optimal threshold, and hence, the delta method cannot be used to obtain a confidence interval; e.g., when Student-*t* distributions are used.

Generally, when an appropriate distribution is found to model the biomarker (good agreement between the empirical and the modeled cumulative distribution functions), this distribution should be used, combined with Bayesian inference to obtain an estimate and a credible interval of the optimal threshold. According to our simulations, when there is no appropriate distribution and the empirical distribution function is unimodal and symmetric, a Student-*t* distribution is a good option. When a distribution is not symmetric or when it is assumed that there is a mixture of distributions within a group (owing to the severity of the disease in the diseased group, for example), a mixture of Dirichlet processes seems to be a good option providing the sample size is at least 50 per group. When the sample size is less than 50 per group, there is no specific recommendation; however, a mixture of Dirichlet processes tends to give coverage probabilities closer to 95% than the other methods but not always too close to 95%.

The Bayesian method to estimate the optimal threshold can be criticized for being difficult to implement; however, many tools are now available to perform Bayesian inference, like WinBUGS. Once a sample from the posterior distribution of the parameters of the marker distribution in each group has been drawn, the computation of the posterior distribution of the optimal threshold becomes straightforward.

In the present simulations, the *NB*/*NC* ratio was arbitrarily fixed to one and the prevalence to 0.5. The optimal threshold being the intersection between the probability density curves of the marker multiplied by *R* in the non-diseased group, a change in *NB*/*NC* or in prevalence would only change the shapes of the curves whose intersection has to be found. For brevity, the results were presented only for several variances in the diseased and non-diseased groups and for various biomarker distributions; changing the variances of the distributions or the distributions themselves is equivalent to changing the shape of the curves. Anyway, it is agreed that the estimation of the *NB*/*NC* value is a difficult task that requires discussions with the physicians and even the patients, and maybe information elicitation, but this is not the subject of the present article. Moreover, the proposed method does not take into account the uncertainty about the prevalence and the *NB*/*NC* value. From the sample size of the study from which the prevalence estimate comes, one can sample *K* values in the posterior distribution of the prevalence and use these values to calculate the *K* Youden functions. This takes into account the statistical uncertainty about the prevalence. When *NB*/*NC* values are obtained from experts, one can use the distribution of these values and sample *K NB*/*NC* values from this distribution. This takes into account the uncertainty about the *NB*/*NC* value when the expert values are relatively close but not exactly equal. When the experts give very different *NB*/*NC* values, it is better to estimate one threshold for each *NB*/*NC* value.

Non-informative priors were used for the present examples and simulations but informative priors can be used in case of prior knowledge on the marker distributions. This is particularly useful for moderate-size data samples.

More flexible distributions could have been used instead of a mixture of Dirichlet processes, such as Polya trees [22], but these distributions would have increased the complexity of the calculations. Moreover, the mixture of Dirichlet processes is very common in Bayesian inference with semi-parametric models [23,24]. Extensions to skewed Student-*t* –or normal– distributions are possible [25]. The literature dedicated to the optimal threshold has proposed methods that deal with measurement errors [26], markers with mass at zero [27], or detection thresholds [8]. The present methods deal with heterogeneity and can be extended to take into account the three latter problems in the inference on the parameters of the biomarker distribution.

The present work may be extended to the estimation of the optimal threshold of a risk-score stemming from a prediction model using methods similar to the ones presented by Subtil and Rabilloud [9]. Flexible distributions (Student-*t* distribution or mixture of Dirichlet processes) would make easier the modelling of a risk-score distribution in the diseased and non-diseased groups.

## Conclusions

The major contribution of the present article was the consideration of the natural heterogeneity of marker measurements between subjects in estimating the optimal threshold of a biomarker with a complex distribution. Whereas this is difficult to carry out with standard frequentist methods, (especially regarding a reliable confidence interval), the Bayesian paradigm seems particularly suitable. The Student-*t* distribution and the mixture of Dirichlet processes are useful distributions in case of heterogeneity in the mean or variance of the biomarker measurements, or in case of outlying values. It is hoped that with a method applicable to a very wide range of biomarkers, more rational approaches will now be used to estimate correctly the optimal threshold of interesting biomarkers.

## Competing interests

The authors declare that they have no conflict of interest.

## Authors’ contributions

FS and MR developed the methods proposed in the manuscript. FS performed the simulations and the data analysis. FS and MR drafted the manuscript. Both authors read and approved the final manuscript.

## Pre-publication history

The pre-publication history for this paper can be accessed here:

http://www.biomedcentral.com/1472-6947/14/53/prepub

## Supplementary Material

Additional file 1 **WinBUGS and R codes for biomarkers modeled with a Student-*t *** **distribution.** WinBUGS code to sample from the posterior distribution of the parameters of a Student-*t* distribution, and R function to sample from the posterior distribution of the optimal threshold of a marker following a Student-*t* distribution in the diseased and non-diseased groups. Click here for file

Additional file 2 **Additional simulation results.** Relative bias of the optimal threshold assuming a Gaussian or a Student-*t* distribution with the coverage probability and the mean width for Design 1 (unequal number of subjects in diseased and non-diseased subjects). Relative bias of the optimal threshold using the empirical, boxcox, and kernel methods, with their associated bootstrap 95% confidence intervals. Click here for file

Additional file 3 Parameterization of the mixture of Dirichlet processes model and sampling techniques. Click here for file

Additional file 4 **Additional results for simulation Designs 1, 2, 3 and 4.** Left and right coverage probability of the credible interval assuming a Gaussian or a Student-*t* distribution for simulation Designs 1, 2, 3, and a Gaussian or a mixture of Dirichlet processes for simulation Design 4, with the associated theoretical threshold as well as Youden index. Click here for file
