# Supplementary material for: Estimating the optimal threshold for a diagnostic biomarker in case of complex biomarker distributions
Source: BMC Med Inform Decis Mak. 2014 Jun 14;14:53. doi: 10.1186/1472-6947-14-53 (PMC4062774; doi:10.1186/1472-6947-14-53)
Supplement: Additional file 1 — WinBUGS and R codes for biomarkers modeled with a Student- t distribution. WinBUGS code to sample from the posterior distribution of the parameters of a Student-t distribution, and R function to sample from the posterior distribution of the optimal threshold of a marker following a Student-t distribution in the diseased and non-diseased groups. [file 1472-6947-14-53-S1.pdf]

Estimating the optimal threshold for a diagnostic biomarker in case of complex biomarker distributions

Fabien Subtil – Muriel Rabilloud

Additional file 1

Student-*t* distribution

Sampling

The following lines correspond to the WinBUGS code used to sample from the posterior distribution of the parameters of a Student-*t* distribution

```
model
{
	## N: number of marker measurements
	## marker: vector marker measurements of length N
	for(i in 1:N)
	{
		marker[i]~dnorm(mu,tau)
	}
	mu~dnorm(0,0.001)
	tau<-pow(exp(logsigma),-2)
	logsigma~dunif(-10,10)
}
```

Optimal threshold

The following lines correspond to an R function used to sample from the posterior distribution of the optimal threshold of a marker that followings a Student-*t* distribution in the diseased and in the non-diseased groups.

```
## prevalence: prevalence of the disease in the population
## NBNC: net benefit - net cost ratio

## The following vectors are sample (of length nbsample) from the posterior
distribution of the parameters of the Student-t distribution in the
diseased and non-diseased groups, obtained using for example WinBUGS:
## marker_dis~t(mu_dis,sigma_dis,nu_dis)
## marker_ndis~t(mu_ndis,sigma_ndis,nu_ndis)
## init: initial value for the optimal threshold, used in the Newton
Raphson maximisation

## the function return a sample of length nbsample from the posterior
distribution of the optimal threshold
```

```
f_threshold_distribution=function(prevalence,NBNC,mu_dis,sigma_dis,nu_dis,m
u_ndis,sigma_ndis,nu_ndis,nbsample,init)
{

      R=(1-prevalence)/prevalence/NBNC


      ## utility function by iteration
      f_util=function(x,mu_dis_i,sigma_dis_i,nu_dis_i,mu_ndis_i,sigma_ndis_
i,nu_ndis_i,R)
      {
            return(-((1-pt((x-mu_dis_i)/sigma_dis_i,df=nu_dis_i))+pt((x-
mu_ndis_i)/sigma_ndis_i,df=nu_ndis_i)*R))
      }

      ## maximisation of the utility function, at iteration i
      f_optidis_threshold=function(i)
      {
            mu_dis_i=mu_dis[i]
            sigma_dis_i=sigma_dis[i]
            nu_dis_i=nu_dis[i]
            mu_ndis_i=mu_ndis[i]
            sigma_ndis_i=sigma_ndis[i]
            nu_ndis_i=nu_ndis[i]


      seuil=nlm(f_util,p=init,mu_dis_i=mu_dis_i,sigma_dis_i=sigma_dis_i,nu_
dis_i=nu_dis_i,

mu_ndis_i=mu_ndis_i,sigma_ndis_i=sigma_ndis_i,nu_ndis_i=nu_ndis_i,R=R)$esti
mate
      }

      ## posterior distribution of the optimal threshold
      threshold_distribution=sapply(1:nbsample,f_optidis_threshold)
      return(threshold_distribution)
}
```
