# Supplementary material for: Estimating the optimal threshold for a diagnostic biomarker in case of complex biomarker distributions
Source: BMC Med Inform Decis Mak. 2014 Jun 14;14:53. doi: 10.1186/1472-6947-14-53 (PMC4062774; doi:10.1186/1472-6947-14-53)
Supplement: Additional file 2 — Additional simulation results. Relative bias of the optimal threshold assuming a Gaussian or a Student-t distribution with the coverage probability and the mean width for Design 1 (unequal number of subjects in diseased and non-diseased subjects). Relative bias of the optimal threshold using the empirical, boxcox, and kernel methods, with their associated bootstrap 95% confidence intervals. [file 1472-6947-14-53-S2.pdf]

Estimating the optimal threshold for a diagnostic biomarker in case of complex biomarker distributions

Fabien Subtil – Muriel Rabilloud

Additional file 2

Part 1 - Complementary simulations or results relative to section "Heterogeneity in the variance of biomarker measurements"

Relative bias of the optimal threshold assuming a Gaussian or a Student-*t* distribution with the coverage probability and the mean width of the 95% credible interval (unequal number of subjects in diseased and non-diseased groups, Design 1)

				Relative bias *						Coverage probability†				CI mean width †			
				Mode		Median		Mean		Quantile		HPD		Quantile		HPD	
N_0	N_1	σ_0	σ_1	Gauss	*t*	Gauss	*t*	Gauss	*t*	Gauss	*t*	Gauss	*t*	Gauss	*t*	Gauss	*t*
100	50	0.07	0.07	-0.00115	-0.00343	-0.00115	-0.00378	-0.00115	-0.00363	0.948	0.948	0.947	0.948	0.017	0.019	0.017	0.019
100	50	0.05	0.05	-0.00091	-0.00333	-0.00094	-0.00366	-0.00092	-0.00352	0.947	0.948	0.942	0.945	0.017	0.019	0.017	0.018
100	50	0.03	0.03	-0.00046	-0.00196	-0.00031	-0.00194	-0.00035	-0.00196	0.947	0.949	0.942	0.946	0.013	0.014	0.013	0.014
100	75	0.07	0.07	-0.00058	-0.00120	-0.00071	-0.00134	-0.00068	-0.00131	0.953	0.954	0.952	0.953	0.024	0.023	0.024	0.023
100	75	0.05	0.05	-0.00059	-0.00132	-0.00050	-0.00145	-0.00051	-0.00140	0.949	0.954	0.945	0.952	0.015	0.016	0.015	0.016
100	75	0.03	0.03	-0.00010	-0.00070	-0.00007	-0.00068	-0.00008	-0.00068	0.954	0.960	0.950	0.955	0.011	0.012	0.011	0.012

True marker distribution in the N_0 non-diseased subjects: Gaussian distribution (mean = -0.3, standard deviation = σ_0)
True marker distribution in the N_1 diseased subjects: Gaussian distribution (mean = -0.25, standard deviation = σ_1)
* Relative bias of the mode, the median, and the mean estimates of the optimal threshold.
† Coverage probability and credible interval (CI) mean width found with the quantile and the Highest Posterior Density (HPD) region method.
Gauss: Gaussian distribution – *t*: Student-*t* distribution.

Relative bias of the optimal threshold using the empirical, boxcox, and kernel methods, with the coverage probability and the mean width of the 95% credible interval (Design 1)

				Relative bias *			Coverage probability †			CI mean width †		
N_0	N_1	σ_0	σ_1	Empirical	Boxcox	Kernel	Empirical	Boxcox	Kernel ‡	Empirical	Boxcox	Kernel ‡
100	50	0.07	0.07	-0.0024	-0.0005	0.0003	0.964	0.947	-	0.059	0.023	-
100	50	0.05	0.05	-0.0023	-0.0002	-0.0000	0.958	0.947	-	0.038	0.016	-
100	50	0.03	0.03	-0.0039	-0.0006	-0.0003	0.909	0.946	-	0.022	0.010	-
100	75	0.07	0.07	-0.0034	-0.0013	0.0003	0.959	0.954	-	0.073	0.032	-
100	75	0.05	0.05	-0.0034	0.0002	0.0004	0.944	0.941	-	0.046	0.022	-
100	75	0.03	0.03	-0.0076	-0.0002	-0.0001	0.829	0.936	-	0.026	0.015	-

True marker distribution in the N_0 non-diseased subjects: Gaussian distribution (mean = -0.3, standard deviation = σ_0)

True marker distribution in the N_1 diseased subjects: Gaussian distribution (mean = -0.25, standard deviation = σ_1)

* Relative bias of the mode, the median, and the mean estimates of the optimal threshold.

† Coverage probability and credible interval (CI) mean width found with the quantile and the Highest Posterior Density (HPD) region method.

Relative bias of the optimal threshold using the empirical, boxcox, and kernel methods, with the coverage probability and the mean width of the 95% credible interval (Design 2)

	Relative bias *			Coverage probability †			CI mean width †		
ν	Empirical	Boxcox	Kernel	Empirical	Boxcox	Kernel ‡	Empirical	Boxcox	Kernel ‡
1	0.0035	- §	0.0302	0.965	- §	-	0.052	- §	-
4	-0.0009	0.0281	0.0055	0.972	0.888	-	0.050	0.029	-
8	-0.0016	0.0073	0.0022	0.970	0.935	-	0.050	0.023	-
12	-0.0025	0.0034	0.0009	0.971	0.947	-	0.050	0.022	-

True marker distribution in the non-diseased group: Gaussian distribution (mean = -0.3, standard deviation = 0.05)

True marker distribution in the diseased group: Student-*t* distribution (mean = -0.25, standard deviation = 0.05, ν degrees of freedom)

* Relative bias of the optimal threshold estimate.

† Coverage probability and credible interval (CI) mean width.

‡ Not estimated since time consuming.

§ Not assessed due to the difficulty to estimate the boxcox transformation parameter for this example.

Relative bias of the optimal threshold using the empirical, boxcox, and kernel methods, with the coverage probability and the mean width of the 95% credible interval (Design 3)

		Relative bias *			Coverage probability †			CI mean width †		
σ_2	p	Empirical	Boxcox	Kernel	Empirical	Boxcox	Kernel ‡	Empirical	Boxcox	Kernel ‡
0.10	0.3	-0.0029	0.0165	0.0059	0.971	0.930	-	0.054	0.028	-
0.10	0.2	-0.0037	0.0106	0.0026	0.970	0.940	-	0.053	0.026	-
0.10	0.1	-0.0016	0.0048	0.0014	0.971	0.942	-	0.052	0.023	-
0.075	0.3	-0.0030	-0.0006	0.0025	0.970	0.946	0.850	0.054	0.024	0.031
0.075	0.2	-0.0020	-0.0004	0.0019	0.970	0.946	0.849	0.052	0.022	0.029
0.075	0.1	-0.0005	-0.0003	0.0018	0.967	0.947	0.842	0.051	0.021	0.028

True marker distribution in the non-diseased group: Gaussian distribution (mean = -0.3, standard deviation = 0.05)

True marker distribution in the diseased group: mixture of two Gaussian distributions $\left(p\times N\left(-0.25,\sigma_2{}^2\right)+\left(1-p\right)\times N\left(-0.25,0.05^2\right)\right)$

* Relative bias of the optimal threshold estimate.

† Coverage probability and credible interval (CI) mean width.

‡ Not estimated since time consuming.

Part 2 - Complementary simulations or results relative to section "Heterogeneity in the mean and the variance of biomarker measurements"

Relative bias of the optimal threshold using the empirical, boxcox, and kernel methods, with the coverage probability and the mean width of the 95% credible interval (Design 4)

			Relative bias *			Coverage probability †			CI mean width †		
N	σ_1	σ_2	Empirical	Boxcox	Kernel	Empirical	Boxcox	Kernel ‡	Empirical	Boxcox	Kernel ‡
200	0.07	0.07	-0.0071	-0.0728	0.0662	0.971	0.290	-	0.092	0.025	-
200	0.08	0.05	0.0394	0.0467	0.1493	0.983	0.630	-	0.111	0.025	-
200	0.10	0.05	0.0289	0.0432	0.1397	0.984	0.685	-	0.103	0.025	-
100	0.07	0.07	-0.0133	-0.0741	0.0768	0.963	0.597	-	0.106	0.035	-
100	0.08	0.05	0.0398	0.0465	0.1658	0.982	0.857	-	0.122	0.035	-
100	0.10	0.05	0.0294	0.0413	0.1554	0.985	0.886	-	0.115	0.035	-
50	0.07	0.07	-0.0312	-0.0750	0.0879	0.937	0.777	-	0.116	0.049	-
50	0.08	0.05	0.0343	0.0451	0.1803	0.980	0.945	-	0.128	0.049	-
50	0.10	0.05	0.0268	0.0400	0.1723	0.980	0.960	-	0.123	0.049	-
30	0.07	0.07	-0.0490	-0.0743	0.0968	0.909	0.864	-	0.122	0.064	-
30	0.08	0.05	0.0224	0.0424	0.1905	0.972	0.970	-	0.127	0.063	-
30	0.10	0.05	0.0194	0.0384	0.1807	0.969	0.974	-	0.126	0.063	-

True marker distribution in the non-diseased group: Gaussian distribution (mean = -0.3, standard deviation = 0.07)
True marker distribution in the diseased group: mixture of two Gaussian distributions $\left(0.5\times N\left(0.05,\sigma_1^{\,2}\right)+0.5\times N\left(-0.25,\sigma_2^{\,2}\right)\right)$

* Relative bias of the optimal threshold estimate. † Coverage probability and credible interval (CI) mean width. ‡ Not estimated since time consuming.
N: number of subjects in each group.
