# Supplementary material for: Estimating the optimal threshold for a diagnostic biomarker in case of complex biomarker distributions
Source: BMC Med Inform Decis Mak. 2014 Jun 14;14:53. doi: 10.1186/1472-6947-14-53 (PMC4062774; doi:10.1186/1472-6947-14-53)
Supplement: Additional file 3 — Parameterization of the mixture of Dirichlet processes model and sampling techniques. [file 1472-6947-14-53-S3.pdf]

Estimating the optimal threshold for a diagnostic biomarker in case of complex biomarker distributions

Fabien Subtil – Muriel Rabilloud

Additional file 3

Mixture of Dirichlet processes

Parameterization

$$
\begin{aligned}
y_i &\sim N\left(\phi_i, \sigma_i^2\right) \\
\left(\phi_i, \sigma_i^2\right) &\sim G \\
G &\sim DP\left(M, G_0\right) \\
G_0 &\sim N\left(\mu_0, \sigma_0^2\right) \times \text{Inv}-\text{Gamma}\left(0.001, 0.001\right) \\
M &\sim \text{Gamma}\left(1,1\right) \\
\mu_0 &\sim N\left(0,1000\right) \\
\sigma_0^2 &\sim \text{Inv}-\text{Gamma}\left(0.001, 0.001\right)
\end{aligned}
$$

y_i denotes the marker measurement in patient i.

Sampling

Gibbs sampling was used to sample from the posterior distributions of the parameters of the mixture of Dirichlet processes in diseased and non-diseased groups. 3000 samples from the posterior distributions were kept. The Youden functions were calculated for each sample and maximized using the Newton-Raphson algorithm. The sample of maxima obtained over all the Youden functions forms the posterior distribution of the optimal threshold.

The `DPdensity` function of package `DPpackage` (Jara A, E. Hanson E, F FQ, Mueller P, Rosner GL. Bayesian non- and semi-parametric modelling in R. 2009, Pontificia Universidad Católica de Chile) under `R` can be used to sample from the posterior distribution of a mixture of Dirichlet processes, but it uses a different parameterization of the mixture than the one presented in the article:

$$
\begin{aligned}
y_i &\sim N\left(\phi_i, \sigma_i^2\right) \\
\left(\phi_i, \sigma_i^2\right) &\sim G \\
G &\sim DP\left(M, G_0\right) \\
G_0 &\sim N\left(\mu_0, \frac{1}{k_0}\sigma^2\right) \times \text{Inv} - \text{Wishart}\left(\nu_1, \psi_1\right) \\
M &\sim \text{Gamma}\left(a_0, b_0\right) \\
k_0 &\sim \text{Gamma}\left(\frac{\tau_1}{2}, \frac{\tau_2}{2}\right) \\
\mu_0 &\sim N\left(m_2, s_2\right) \\
\psi_1 &\sim \text{Inv} - \text{Wishart}\left(\nu_2, \psi_2\right)
\end{aligned}
$$

An R code is provided at the end of the file to calculate the posterior distribution of a biomarker threshold when this biomarker is modelled according to a mixture of Dirichlet processes in diseased and non-diseased groups, using the DPpackage.

In the cancer of the upper aerodigestive tract application, using the following parameters values, the optimal threshold obtained is very similar to the one obtained using the first proposed parameterization:
$\nu_1 = 4, a_0 = b_0 = 1, \tau_1 = \tau_2 = 1, m_2 = 0, s_2 = 1000, \nu_2 = 4, \psi_2 = 5$

The user has to specify the vectors or parameters colored in blue in the code.

```
library(DPpackage)


##********** PARAMETERS TO BE DEFINED BEFORE MCMC CALCULATIONS **********##
y_dis= ## vector of marker measurements in the diseased group
y_ndis= ## vector of marker measurements in the non-diseased group
start_threshold= ## starting value for the optimal threshold in the Newton-Raphson algorithm
prevalence= ## prevalence of the diseased in the population
NBNC= ## net benefit / net cost ratio

R=(1-prevalence)/prevalence/NBNC


##*********** DIRICHLET PROCESS MIXTURE FOR DISEASED PATIENTS ***********##
## ---- PRIORS
nu1_dis=4
prior_dis=list(a0=1,b0=1,m2=rep(0,1),s2=diag(1000,1),psiinv2=solve(diag(5,1)),nu1=nu1_dis,nu2=4,tau1=1,tau2=1)

## ---- PARAMETERS FOR SIMULATION
nburn=5000 ## number of iterations discarded
nsave=500 ## number of iterations to keep
nskip=1 ## thining parameter
ndisplay=100
statex=NULL
mcmc=list(nburn=nburn,nsave=nsave,nskip=nskip,ndisplay=ndisplay)
```

```
## ---- DIRICHLET PROCESSE MIXTURE
fit_dis=DPdensity(y=y_dis,prior=prior_dis,mcmc=mcmc,state=statex,status=TRU
E)


##********* DIRICHLET PROCESS MIXTURE FOR NON DISEASED PATIENTS ********##
## ---- PRIORS
nu1_ndis=4
prior_ndis=list(a0=1,b0=1,m2=rep(0,1),s2=diag(1000,1),psiinv2=solve(diag(5,
1)),nu1=nu1_ndis,nu2=4,tau1=1,tau2=1)

## ---- DIRICHLET PROCESS MIXTURE
fit_ndis=DPdensity(y=y_ndis,prior=prior_ndis,mcmc=mcmc,state=statex,status=
TRUE)


##*********  POSTERIOR DISTRIBUTION OF THE OPTIMAL THRESHOLD ***********##

## function to maximize the utility function at iteration iter from the
mcmc
f_semipara=function(iter,fit_dis,fit_ndis,y_dis,y_ndis,R,nu1_dis,nu1_ndis,s
tart_threshold)
{

      ## parameters for the non-diseased group
      nbcluster0=fit_ndis$save.state$thetasave[iter,4]
      list_clust_phi0=fit_ndis$save.state$randsave[iter,seq(1,length(y_ndis
),by=2)]
      list_clust_var0=fit_ndis$save.state$randsave[iter,seq(2,length(y_ndis
),by=2)]
      tab_clust_phi0=table(list_clust_phi0)
      clusternbpatient0=as.numeric(tab_clust_phi0)
      phi0=unique(list_clust_phi0)
      vari0=(unique(list_clust_var0))[order(phi0)]
      phi0=sort(phi0)
      M0=fit_ndis$save.state$thetasave[iter,5]
      m0=fit_ndis$save.state$thetasave[iter,1]
      k0=fit_ndis$save.state$thetasave[iter,2]
      psi0=fit_ndis$save.state$thetasave[iter,3]

      ## parameters for the diseased group
      nbcluster1=fit_dis$save.state$thetasave[iter,4]
      list_clust_phi1=fit_dis$save.state$randsave[iter,seq(1,length(y_dis),
by=2)]
      list_clust_var1=fit_dis$save.state$randsave[iter,seq(2,length(y_dis),
by=2)]
      tab_clust_phi1=table(list_clust_phi1)
      clusternbpatient1=as.numeric(tab_clust_phi1)
      phi1=unique(list_clust_phi1)
      vari1=(unique(list_clust_var1))[order(phi1)]
      phi1=sort(phi1)
      M1=fit_dis$save.state$thetasave[iter,5]
      m1=fit_dis$save.state$thetasave[iter,1]
      k1=fit_dis$save.state$thetasave[iter,2]
      psi1=fit_dis$save.state$thetasave[iter,3]
```

```
      ## maximization of the utility function
      return(nlm(f_util,p=start_threshold,clusternbpatient1,phi1,vari1,k1,M
1,m1,psi1,clusternbpatient0,phi0,vari0,k0,M0,m0,psi0,R,nu1_dis,nu1_ndis,len
gth(y_dis),length(y_ndis))$estimate)
}


## utility function to be maximized
f_util=function(x,clusternbpatient1,phi1,vari1,k1,M1,m1,psi1,clusternbpatie
nt0,phi0,vari0,k0,M0,m0,psi0,R,nu1_dis,nu1_ndis,nb_dis,nb_ndis)
{
      return(-((1-
sum((clusternbpatient1*(pnorm(x,phi1,sqrt(vari1))))+M1*(pt((x-
m1)/sqrt((1+1/k1)*psi1/nu1_dis),df=nu1_dis))))/(M1+nb_dis)+

(sum(clusternbpatient0*(pnorm(x,phi0,sqrt(vari0))))+M0*pt((x-
m0)/sqrt((1+1/k0)*psi0/nu1_ndis),df=nu1_ndis))/(M0+nb_ndis)*R))
}


## optimal threshold posterior distribution
threshold_posterior=sapply(1:nsave,f_semipara,fit_dis,fit_ndis,y_dis,y_ndis
,R,nu1_dis,nu1_ndis,start_threshold)
```
