# Supplementary material for: Estimating the optimal threshold for a diagnostic biomarker in case of complex biomarker distributions
Source: BMC Med Inform Decis Mak. 2014 Jun 14;14:53. doi: 10.1186/1472-6947-14-53 (PMC4062774; doi:10.1186/1472-6947-14-53)
Supplement: Additional file 4 — Additional results for simulation Designs 1, 2, 3 and 4. Left and right coverage probability of the credible interval assuming a Gaussian or a Student-t distribution for simulation Designs 1, 2, 3, and a Gaussian or a mixture of Dirichlet processes for simulation Design 4, with the associated theoretical threshold as well as Youden index. [file 1472-6947-14-53-S4.pdf]

Estimating the optimal threshold for a diagnostic biomarker in case of complex biomarker distributions

Fabien Subtil – Muriel Rabilloud

Additional file 4

Left and right coverage probabilities of the credible interval assuming a Gaussian or a Student-*t* distribution, with the associated theoretical threshold as well as Youden index (Design 1)

						Quantile				HPD			
						Gauss		*t*		Gauss		*t*	
N_0	N_1	σ_0	σ_1	Theoretical optimal threshold	Youden index	1 – LCP *	1 – RCP *	1 – LCP *	1 – RCP *	1 – LCP *	1 – RCP *	1 – LCP *	1 – RCP *
100	100	0.07	0.07	-0.250	1.525	0.030	0.025	0.028	0.025	0.030	0.027	0.029	0.026
100	100	0.05	0.05	-0.250	1.683	0.027	0.026	0.026	0.023	0.028	0.028	0.030	0.026
100	100	0.03	0.03	-0.250	1.904	0.024	0.028	0.023	0.022	0.025	0.030	0.025	0.024
50	50	0.07	0.07	-0.250	1.525	0.028	0.022	0.025	0.021	0.029	0.023	0.026	0.024
50	50	0.05	0.05	-0.250	1.683	0.024	0.026	0.024	0.025	0.024	0.027	0.026	0.026
50	50	0.03	0.03	-0.250	1.904	0.029	0.026	0.036	0.023	0.031	0.029	0.036	0.024
100	100	0.07	0.03	-0.250	1.717	0.024	0.026	0.053	0.007	0.027	0.023	0.059	0.006
100	100	0.03	0.07	-0.250	1.717	0.026	0.028	0.007	0.055	0.024	0.030	0.008	0.058
50	50	0.07	0.03	-0.250	1.717	0.022	0.030	0.046	0.011	0.027	0.027	0.050	0.010
50	50	0.03	0.07	-0.250	1.717	0.025	0.022	0.006	0.046	0.023	0.029	0.006	0.052
100	50	0.07	0.07	-0.250	1.525	0.023	0.029	0.017	0.035	0.025	0.028	0.017	0.035
100	50	0.05	0.05	-0.250	1.683	0.024	0.029	0.015	0.037	0.026	0.032	0.018	0.037
100	50	0.03	0.03	-0.250	1.904	0.026	0.027	0.017	0.035	0.027	0.030	0.019	0.036
100	75	0.07	0.07	-0.250	1.525	0.023	0.024	0.019	0.026	0.023	0.025	0.019	0.028
100	75	0.05	0.05	-0.250	1.683	0.022	0.029	0.018	0.028	0.024	0.031	0.019	0.029
100	75	0.03	0.03	-0.250	1.904	0.025	0.021	0.020	0.020	0.027	0.023	0.023	0.023

True marker distribution in the N_0 non-diseased subjects: Gaussian distribution (mean = -0.3, standard deviation = σ_0)

True marker distribution in the N_1 diseased subjects: Gaussian distribution (mean = -0.25, standard deviation = σ_1)

* LCP: Left Coverage Probability – RCP: Right Coverage Probability

Gauss: Gaussian distribution – *t*: Student-*t* distribution.

Left and right coverage probabilities of the credible interval assuming a Gaussian or a Student-*t* distribution, with the associated theoretical threshold as well as Youden index (Design 2)

			Quantile				HPD			
			Gauss		*t*		Gauss		*t*	
ν	Theoretical optimal threshold	Youden index	1 – LCP *	1 – RCP *	1 – LCP *	1 – RCP *	1 – LCP *	1 – RCP *	1 – LCP *	1 – RCP *
1	-0.262	1.351	1.000	0.000	0.051	0.017	1.000	0.000	0.044	0.019
4	-0.271	1.371	0.468	0.001	0.031	0.015	0.468	0.001	0.030	0.017
8	-0.273	1.377	0.123	0.008	0.025	0.023	0.127	0.009	0.025	0.024
12	-0.274	1.379	0.068	0.012	0.024	0.021	0.070	0.012	0.027	0.021

True marker distribution in the non-diseased group: Gaussian distribution (mean = -0.3, standard deviation = 0.05)
True marker distribution in the diseased group: Student-*t* distribution (mean = -0.25, standard deviation = 0.05, ν degrees of freedom)
* LCP: Left Coverage Probability – RCP: Right Coverage Probability

Gauss: Gaussian distribution – *t*: Student-*t* distribution.

Left and right coverage probabilities of the credible interval assuming a Gaussian or a Student-*t* distribution, with the associated theoretical threshold as well as Youden index (Design 3)

				Quantile				HPD			
				Gauss		*t*		Gauss		*t*	
σ_2	p	Theoretical optimal threshold	Youden index	1 – LCP *	1 – RCP *	1 – LCP *	1 – RCP *	1 – LCP *	1 – RCP *	1 – LCP *	1 – RCP *
0.10	0.3	-0.267	1.359	0.263	0.001	0.022	0.022	0.264	0.002	0.023	0.027
0.10	0.2	-0.270	1.366	0.193	0.003	0.021	0.023	0.192	0.004	0.021	0.025
0.10	0.1	-0.273	1.374	0.102	0.009	0.021	0.020	0.104	0.010	0.022	0.023
0.075	0.3	-0.270	1.366	0.064	0.013	0.018	0.029	0.064	0.013	0.019	0.028
0.075	0.2	-0.272	1.371	0.050	0.015	0.020	0.028	0.050	0.015	0.019	0.029
0.075	0.1	-0.274	1.377	0.036	0.017	0.019	0.027	0.039	0.019	0.020	0.026

True marker distribution in the non-diseased group: Gaussian distribution (mean = -0.3, standard deviation = 0.05)
True marker distribution in the diseased group: mixture of two Gaussian distributions $\left(p \times N\left(-0.25, {\sigma_2}^2\right) + (1-p) \times N\left(-0.25, 0.05^2\right)\right)$

[*] LCP: Left Coverage Probability – RCP: Right Coverage Probability

Gauss: Gaussian distribution – *t*: Student-*t* distribution.

Left and right coverage probabilities of the credible interval assuming a Gaussian distribution or a Dirichlet process mixture, with the associated theoretical threshold as well as Youden index (Design 4)

					Quantile				HPD			
					Gauss		Dirichlet		Gauss		Dirichlet	
N	σ_1	σ_2	Theoretical optimal threshold	Youden index	1 – LCP *	1 – RCP *	1 – LCP *	1 – RCP *	1 – LCP *	1 – RCP *	1 – LCP *	1 – RCP *
200	0.07	0.07	-0.207	1.543	0.377	0.000	0.010	0.043	0.369	0.000	0.012	0.062
200	0.08	0.05	-0.239	1.515	1.000	0.000	0.012	0.032	1.000	0.000	0.012	0.046
200	0.10	0.05	-0.240	1.514	1.000	0.000	0.012	0.029	1.000	0.000	0.012	0.043
100	0.07	0.07	-0.207	1.543	0.207	0.001	0.007	0.049	0.201	0.001	0.009	0.069
100	0.08	0.05	-0.239	1.515	1.000	0.000	0.013	0.028	1.000	0.000	0.011	0.042
100	0.10	0.05	-0.240	1.514	1.000	0.000	0.011	0.029	1.000	0.000	0.012	0.043
50	0.07	0.07	-0.207	1.543	0.119	0.002	0.004	0.025	0.113	0.003	0.004	0.044
50	0.08	0.05	-0.239	1.515	0.983	0.000	0.016	0.015	0.980	0.000	0.014	0.026
50	0.10	0.05	-0.240	1.514	0.988	0.000	0.023	0.014	0.985	0.000	0.019	0.019
30	0.07	0.07	-0.207	1.543	0.074	0.003	0.006	0.012	0.065	0.006	0.005	0.021
30	0.08	0.05	-0.239	1.515	0.889	0.000	0.037	0.002	0.873	0.000	0.032	0.005
30	0.10	0.05	-0.240	1.514	0.909	0.000	0.053	0.002	0.896	0.000	0.045	0.004

True marker distribution in the non-diseased group: Gaussian distribution (mean = -0.3, standard deviation = 0.07)

True marker distribution in the diseased group: mixture of two Gaussian distributions $\left(0.5\times N\left(0.05,\sigma_1^{\,2}\right)+0.5\times N\left(-0.25,\sigma_2^{\,2}\right)\right)$

* LCP: Left Coverage Probability – RCP: Right Coverage Probability

N: number of subjects in each group – Gauss: Gaussian distribution – Dirichlet: mixture of Dirichlet processes.
